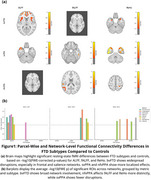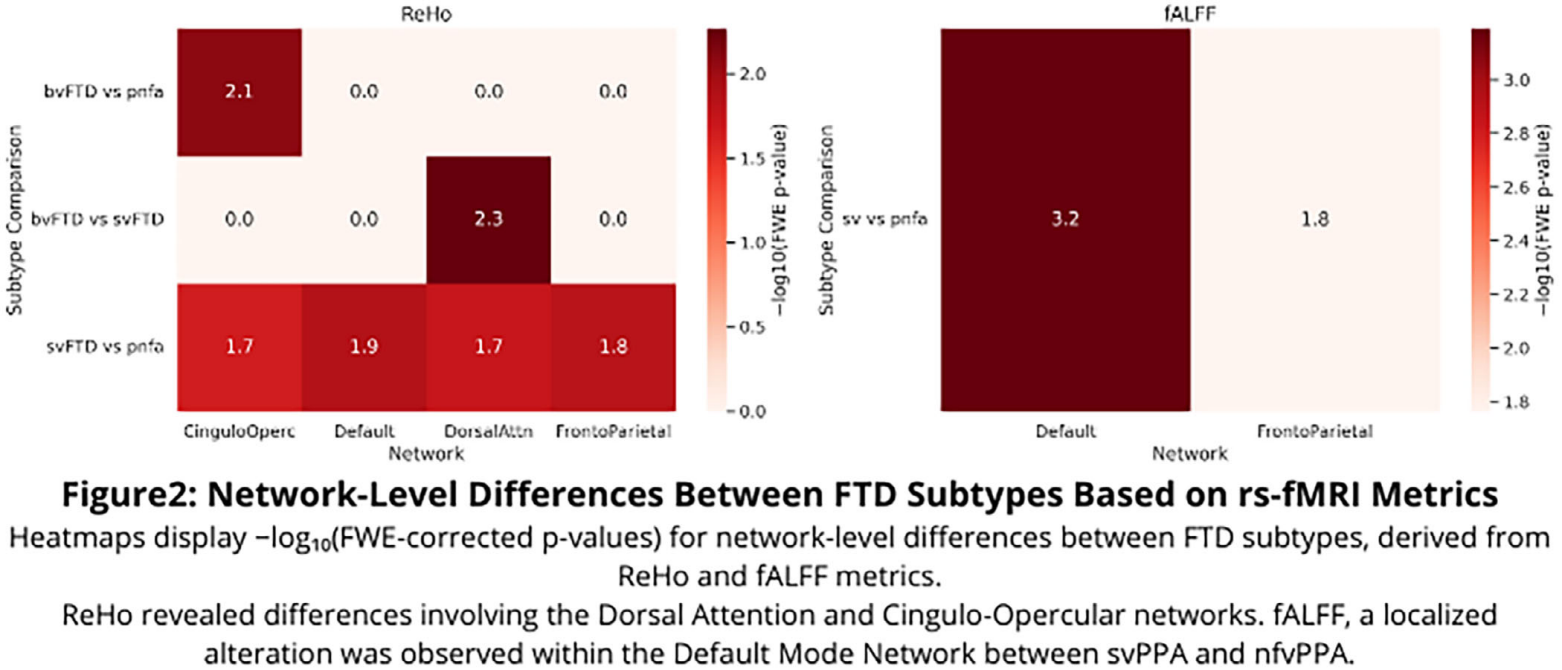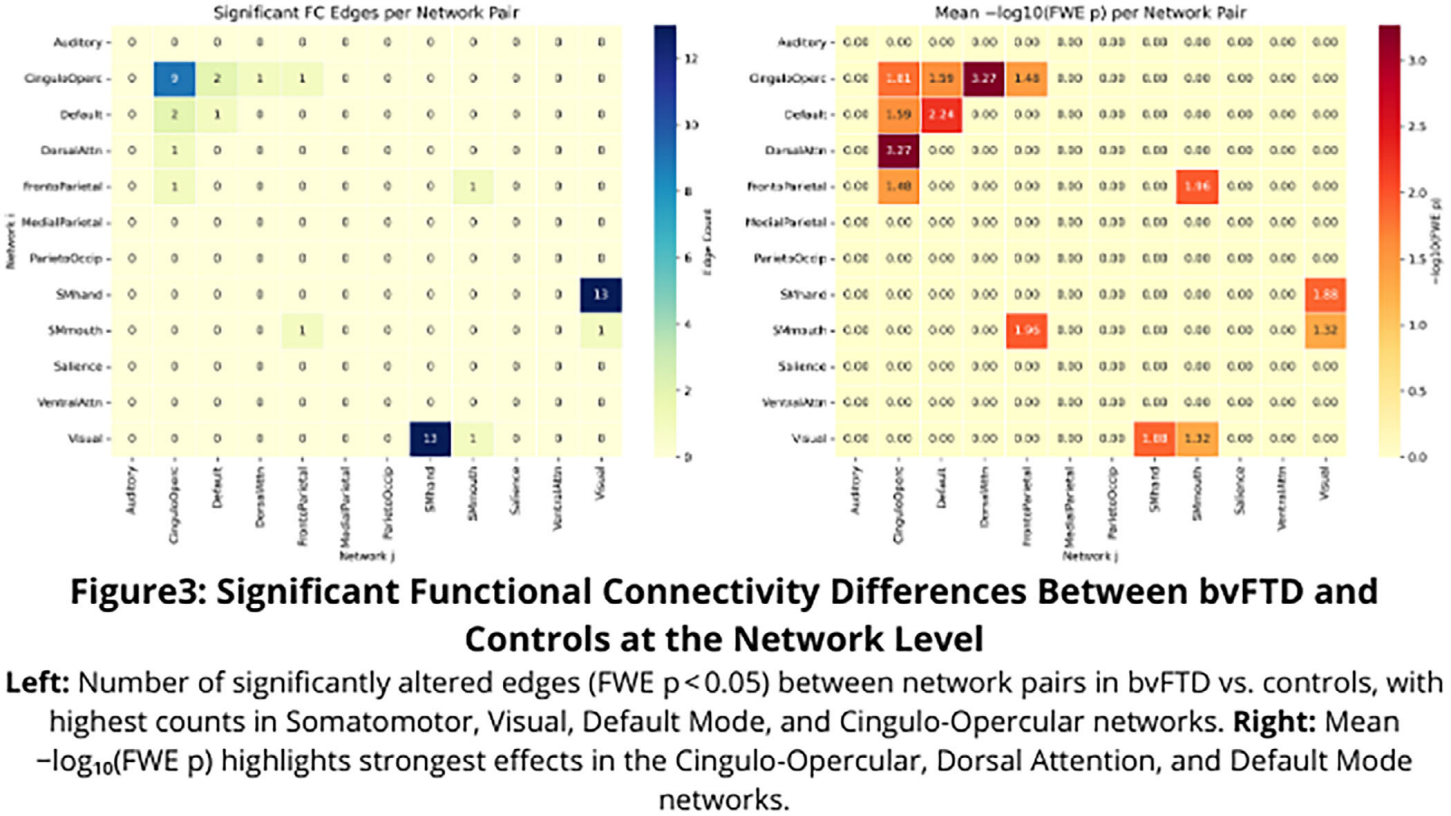# Multimodal Resting‐State fMRI Reveals Subtype‐Specific Network‐Level Functional Differences in Frontotemporal Dementia

**DOI:** 10.1002/alz70861_108581

**Published:** 2025-12-23

**Authors:** Oumayma Soula, Udunna Anazodo, Nawres Khlifa, Ahmed Rebai

**Affiliations:** ^1^ Faculty of medicine of Sfax, University of Sfax, Sfax Tunisia; ^2^ Laboratory of Molecular and Cellular Screening Processes, Centre of Biotechnology of Sfax, University of Sfax, Sfax Tunisia; ^3^ Laboratory of Biophysics and Medical Technologies, Higher Institute of Medical Technologies of Tunis, University Al Manar, Tunis Tunisia; ^4^ Montreal Neurological Institute, McGill University, Montreal, QC Canada; ^5^ Medical Artificial Intelligence (MAI) Laboratory, Crestview Radiology Limited, Lagos Nigeria

## Abstract

**Background:**

Frontotemporal dementia (FTD) is a major cause of early‐onset dementia, including behavioral variant FTD (bvFTD), semantic variant primary progressive aphasia (svPPA), and nonfluent variant PPA (nfvPPA). Each subtype is linked to distinct but overlapping disruptions in brain networks. Resting‐state functional MRI (rs‐fMRI) offers a non‐invasive way to assess intrinsic brain activity and may help identify subtype‐specific functional biomarkers **(1)**. While functional connectivity (FC) has been widely studied, other rs‐fMRI metrics: Amplitude of Low‐Frequency Fluctuations (ALFF), fractional ALFF (fALFF), and Regional Homogeneity (ReHo), remain underexplored. These metrics capture complementary aspects of brain function, and their combined use may improve early and accurate differentiation of FTD subtypes.

**Method:**

We analyzed T1‐weighted and rs‐fMRI data from 98 FTD patients:41 bvFTD (mean age 60.6 ± 6.5 years; 27 males, 14 females), 31 svPPA (63.2 ± 6.4 years; 19 males, 12 females), and 26 nfvPPA (68.0 ± 7.7 years; 12 males, 14 females), along with 88 controls (62.8 ± 7.6 years; 38 males, 50 females), obtained from the NIFD/FTLDNI database via the IDA platform **(2)**. Preprocessing was performed using fMRIPrep **(3)**, followed by XCP‐D **(4)**. ALFF, fALFF, ReHo, and FC metrics. All measures were averaged within Gordon atlas regions **(5)**. Two‐sample t‐tests with Bonferroni correction were used for group comparisons (FWE *p* < 0.05; FC *p* < 0.01), and results were mapped to functional networks.

**Result:**

Significant differences were observed between FTD subtypes and controls (Figure 1), bvFTD showed the most widespread ALFF alterations (p = 0.020), especially in frontal and salience regions. svPPA exhibited localized ALFF changes (p ≈ 0.020). fALFF differences were most notable in nfvPPA (p = 0.0186). ReHo alterations were strongest in svPPA (p = 0.0076), followed by nfvPPA (p = 0.017) and bvFTD (p = 0.027). ReHo and fALFF distinguished svPPA from nfvPPA (Figure 2). Only bvFTD showed significant FC disruptions (29 edges, FWE *p* < 0.05)(Figure 3).

**Conclusion:**

Multimodal rs‐fMRI revealed subtype‐specific alterations in FTD. bvFTD showed widespread disruptions, while svPPA and nfvPPA had more localized changes in attention and default networks.

**Reference**:

1. Canu et al., *Mol Psychiatry*, 2022.

2. https://ida.loni.usc.edu/login.jsp

3. Esteban et al., *Nat Methods*, 2019.

4. Mehta et al., *Imaging Neurosci*, 2024.

5. Gordon et al., *Cereb Cortex*, 2016.